# Evolutionary arms race in ant-ant mimicry: *Camponotus lateralis* lags behind in mimicking color patterns and sizes of regional *Crematogaster* models

**DOI:** 10.1038/s41598-025-25035-y

**Published:** 2025-11-20

**Authors:** Felix Kraker, Herbert C. Wagner

**Affiliations:** Institute of Biology, University of Graz, Universitätsplatz 2/I, 8010 Graz, Austria

**Keywords:** Batesian mimicry, Red-green-blue (RGB) color measurements, *Crematogaster scutellaris*, Chase-away hypothesis, Ecology, Ecology, Evolution, Zoology

## Abstract

Over a century ago, biologists proposed the Mediterranean ant *Camponotus lateralis* mimicked the coloration of the common and unpalatable ant *Crematogaster scutellaris*. A more recent hypothesis suggested that *Ca. lateralis* also mimicked the color of two additional models, *Cr. schmidti* and *Cr. ionia*, in their respective range. This study aims to test the hypothesis using red–green–blue values of 573 model and 957 mimic individuals and whether also size is affected by mimicry. The results support the regional-mimicry hypothesis: *Camponotus lateralis* is phenotypically more similar to syn- than to allotopic models. However, regional mimics evolutionarily lag behind the stronger radiated color and body-size traits of the models. *Camponotus lateralis* mimicked the coloration of the West Mediterranean species *Cr. scutellaris* least accurately, pointing to the possibility that the East Mediterranean species *Cr. schmidti* and *Cr. ionia* are the primary models, and *Cr. scutellaris* entered the system at a later stage. Fascinatingly, the example of *Ca. lateralis* is one of several analogous cases of convergent color evolution in camponotine ants that mimic *Crematogaster* models of different coloration. The unusually strong discriminant power of color variables in *Crematogaster* models—only partially replicated by their mimics—indicates a chase-away dynamic in response to Batesian mimicry.

**Supplementary Information:**

The online version contains supplementary material available at 10.1038/s41598-025-25035-y.

## Introduction

Ants are one of the most abundant arthropod groups in the world and constitute a large proportion of the total biomass in their ecosystems^1,2^. In some habitats, few ant species are especially frequent and exert exceptionally strong ecological influence and dominance^3,4^. However, high abundance increases predation pressure, requiring finely tuned adaptations like strong defensive mechanisms^5^. Some ants contain, for example, formic acid, others rely on their stinger or powerful bite^6,7^. Members of the myrmicine genus *Crematogaster* can be dominant ant species in their habitats, form large colonies^8–10^, and release a contact poison that can kill other ants^11–15^ and repel vertebrate predators^8,16,17^.

Mimicry is an adaptation in which one species evolves to display resemblance (e.g., visually or olfactory) to another. In Batesian mimicry, a harmless species reduces predation by imitating another species of low profitability to be consumed^18–20^. The fact that the majority of Batesian mimics are inaccurate has challenged the science of mimicry^21–25^. Several non-mutually exclusive hypotheses suggest explanations: The most popular one is relaxed selection by predators suffering under information limitation and, thus, avoiding the risk of accidentally attacking a model^26–28^. Another idea is the multiple-model theory, in which a mimic represents a phenotypical compromise intermediate between different models^29–32^. Recently, it has also been suggested that accurate mimicry is an unstable evolutionary state because it can lead to multiple conflicting selective costs outside of mimicry^33–36^. Because of their defensibility and unpalatability, dominant ant species are popular models for Batesian mimetic arthropods^37–41^. *Crematogaster* species serve as models for many mimetic species, including camponotine ants^16,17,42–47^.

*Camponotus lateralis* is a widespread Eurocaucasian-Mediterranean formicine ant species^8,48^, palatable for predators^17^, with small colonies^49^ and timid behavior^50,51^. It often occurs syntopically with species of the *Crematogaster scutellaris* group, with which it has a close interspecific relationship^45,52–56^. *Camponotus lateralis* minor workers (and rarely also major workers^53,57^) follow pheromone trails of *Cr. scutellaris* to colonies of trophobionts and exploit these^45,53,54,57–63^. Similar associations of trail-following behavior have also been observed with *Cr. schmidti* as well as with *Cr. ionia*^8,55,64,65^.

Despite being treated as a single species based on an in-depth morphometric study^66^, *Ca. lateralis* exhibits a substantial geographic variability in coloration. Typically, the head of workers is reddish, and the gaster is blackish. The color of the mesosoma and petiole varies from almost fully blackish to fully reddish^8,64,66–68^. Its color pattern resembles *Cr. scutellaris* sometimes so closely that even taxonomists have confused them^49,53,58^. The adaptive value of this Batesian mimicry lays probably in the reduction of lizard predation. Mediterranean true lizards perceive the color red well^69–72^, consume up to 60% of their total arthropod prey items in the form of ants^17,73–82^, but strongly avoid *Crematogaster scutellaris*^17^. It was hypothesized that *Ca. lateralis* mimics the color patterns of regional occurring *Crematogaster* model-species^8,64,66^: *Crematogaster scutellaris* occurs in the Western Mediterranean, the Apennine Peninsula, Istria, Dalmatia, and Croatian Islands^8,49,67,83–88^; it is blackish with a reddish head. *Crematogaster schmidti* occurs in Friuli, Slovenia, large parts of the Balkan Peninsula, Crimea, Anatolia, and the Caucasus^8,49,83–89^; it has a reddish head, a reddish mesosoma, and a reddish petiole. *Crematogaster ionia* s.l. (comment: it seems possible that *Cr. ionia* represents a cryptic species complex^65,90^) occurs in the southernmost Balkan mainland, on most Greek Islands, in Anatolia, on Cyprus, and its distribution stretches to the southeast until Israel^49,65,86,90–93^; it is homogenously brownish to blackish. So far, the hypothesis of geographical color congruence between *Ca. lateralis* and the three *Crematogaster* model-species has been postulated merely based on the subjective visual assessment of myrmecologists^8,45,64,66^. This study tests the hypothesis via red–green–blue (RGB) color measurements of ant material from 18 study sites from North Italy in the northwest to the Aegean island Karpathos in the southeast and tests whether also body size is affected by mimicry.

Research question 1: Which color or size traits differ between the three *Crematogaster* model-species and between their syntopic *Camponotus lateralis* mimics?

Research question 2: Is *Camponotus lateralis* more similar to syn- than to allotopic models in color and size?

Research question 3: Can *Camponotus lateralis* be correctly allocated to the syntopic model species using color values, and does classification accuracy differ among model species?

## Results

We have collected values of 18 RGB color characters from six body parts: the head, the pronotum, the mesonotum, the propodeum, the petiole, and the gaster (character definitions given under Methods). Three categories of *Camponotus lateralis* were classified by their syntopically occurring *Crematogaster* species and termed „*scutellaris*-syntopic “, „*schmidti*-syntopic”, and „*ionia*-syntopic “.

## Research question 1: Which color or size traits differ between the three *Crematogaster* model-species and between their syntopic* Camponotus lateralis* mimics?

**Fig. 1:**
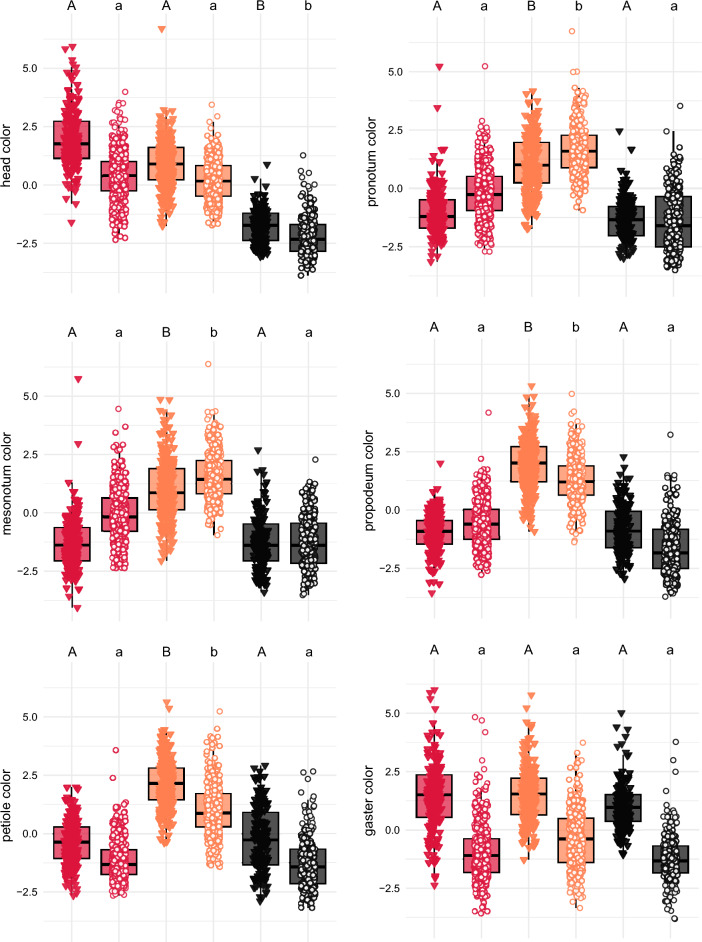
First principal components of RGB color variables of six body parts (head, pronotum, mesonotum, propodeum, petiole, and gaster). Pairwise comparisons between *Crematogaster scutellaris* (*n* = 179; red triangles), *Cr. schmidti* (*n* = 244; orange triangles), and *Cr. ionia* s.l. (*n* = 150; black triangles) as well as between the *scutellaris*-syntopic (*n* = 388; red circles), the *schmidti*-syntopic (*n* = 327; orange circles), and the *ionia*-syntopic (*n* = 242; black circles) *Camponotus lateralis* categories were analyzed via generalized linear mixed models. Different letters above the boxplots show significant differences based after Bonferroni-Holm correction; capital letters are used for *Crematogaster*, lowercase letters for *Ca. lateralis*.

The three color-traits (RGB) of each body part were reduced to principal components, with the first (PC1) explaining large percentages of the total color variance: head: 89.9%; pronotum: 88.6%; mesonotum: 86.9%; propodeum: 89.7%; petiole: 90.8%; and gaster: 91.4%. PC1 of the head differed significantly between *Crematogaster ionia* s.l. and the two other *Crematogaster* species as well as between the *ionia*-syntopic and the two other *Ca. lateralis* categories. In contrast, there were no differences between *Cr. scutellaris* and *Cr. schmidti* as well as between the *scutellaris*-syntopic and *schmidti*-syntopic category of *Ca. lateralis*. The PC1s of the pronotum, the mesonotum, the propodeum, and the petiole in *Cr. schmidti* differed from those of *Cr. scutellaris* and *Cr. ionia* s.l., while there were no differences in any PC1s of the two latter species. Also in the *schmidti*-syntopic *Ca. lateralis* category, all PC1s differed from those of the *scutellaris*-syntopic and *ionia*-syntopic category of *Ca. lateralis*, while there were no differences between the two latter categories. No gaster PC1s differed among *Crematogaster* species or *Ca. lateralis* categories; instead, the variability in this character is mainly explained by genus identity (*Crematogaster* vs. *Camponotus*) (Fig. 1; means and standard variability of color traits in Supplementary material Table S1; pairwise *p*-values for comparisons of the PC1s in Table S2).

**Fig. 2:**
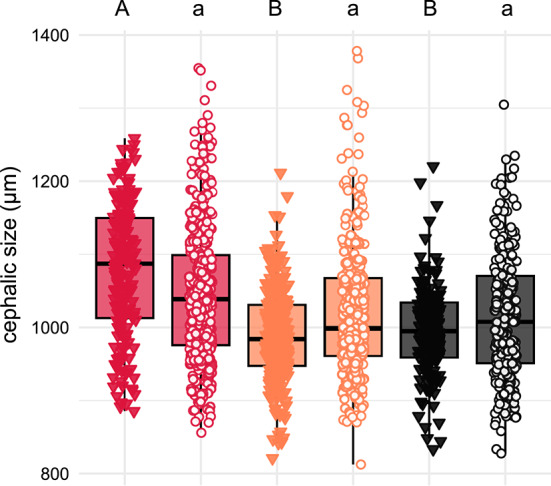
Cephalic size [CS; definition under Table 3] of *Crematogaster scutellaris* (red triangles), *scutellaris*-syntopic *Ca. lateralis* (red circles), *Cr. schmidti* (orange triangles), *schmidti*-syntopic *Ca. lateralis* (orange circles), *Cr. ionia* (black triangles), and *ionia*-syntopic *Ca. lateralis* (black circles). Different letters above the boxplots show significant differences after Bonferroni-Holm correction; capital letters are used for *Crematogaster*, lowercase letters for *Ca. lateralis*.

Cephalic size (CS; the standard measure for body size in ants) was significantly larger in *Cr. scutellaris* (1079 ± 92 µm, *n* = 179), exceeding those of *Cr. schmidti* (990 ± 65 µm, *n* = 244) and *Cr. ionia* s.l. (996 ± 63 µm, *n* = 150) by 8–9%. In contrast, *Cr. schmidti* and *Cr. ionia* s.l. did not differ from each other. Cephalic size of *scutellaris*-syntopic *Camponotus lateralis* minor workers (1047 ± 93 µm, *n* = 388) was 3% larger than in *schmidti*-syntopic (1019 ± 93 µm, *n* = 327) and *ionia*-syntopic (1015 ± 88 µm, *n* = 242) ones, but the differences were not significant. The latter two categories did not differ from each other (Fig. 2).

## Research question 2: Is *Camponotus lateralis* more similar to syn- than to allotopic models in color and size?

**Fig. 3:**
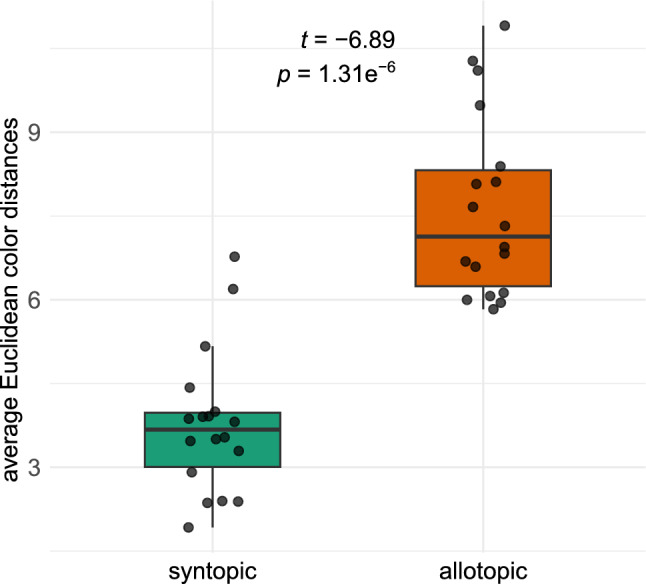
Average Euclidean color distances between nests of *Crematogaster* model-species and of *Camponotus lateralis* in syntopy and in allotopy (one-sided, paired t-test).

Euclidean color distances between *Crematogaster* and *Ca. lateralis* were 3.8 ± 1.3 in syntopy and 7.6 ± 1.6 in allotopy; *Ca. lateralis* was highly significantly more similar to syn- than to allotopic models (Fig. 3). On site 17, a site with *Cr. ionia* s.l. on Crete, the difference between syn- and allotopic model-mimic similarity was highest (7.7), while only on site 7, a site with *Cr. scutellaris* in Dalmatia, the syntopic difference was larger than the allopatric one (0.2; Supplementary material Table S3).

**Fig. 4:**
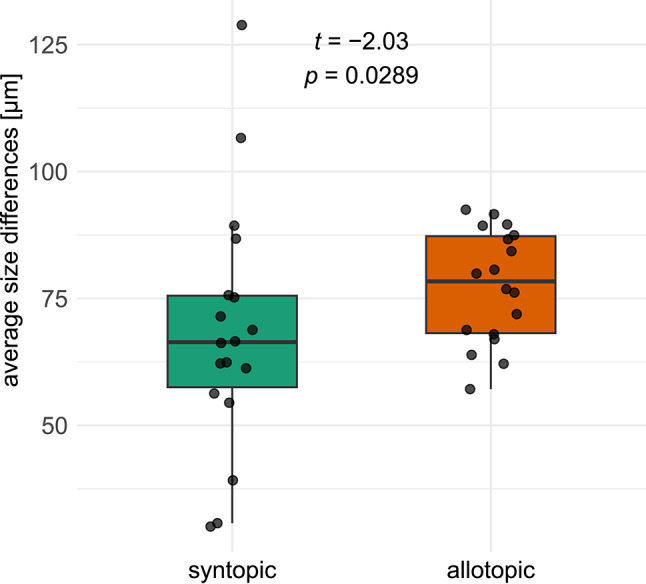
Average size differences between nests of *Crematogaster* model-species and *Camponotus lateralis* in syntopy and in allotopy (one-sided, paired t-test).

Size differences between *Crematogaster* and *Ca. lateralis* were 68 ± 25 μm in syntopy and 77 ± 11 μm in allotopy (Supplementary material Table S4), they were slightly significantly smaller in syn- than in allotopy (Fig. 4).

**Fig. 5:**
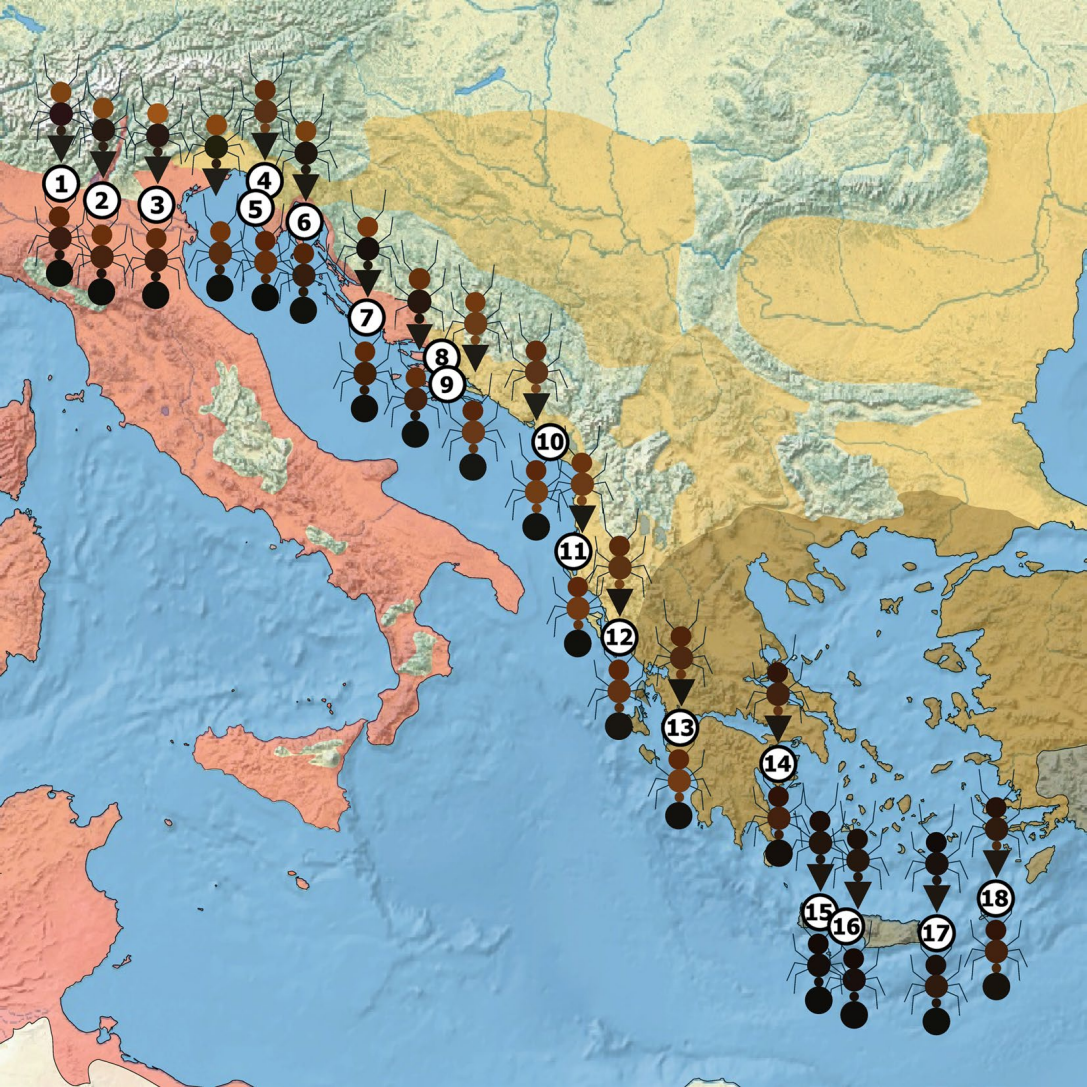
The 18 collection sites and mean empirically-collected color data of models, represented with a triangle-shaped gaster (site 1–3 and 5–8 are *Crematogaster scutellaris*, 4 and 9–13 are *Cr. schmidti* and 14–18 are *Cr. ionia* s.l.), and the mimic, *Camponotus lateralis*, represented with a round gaster. The background colors show the areas of the model species: rose: *Cr. scutellaris*; yellow: *Cr. schmidti*; grey: *Cr. ionia* s.l.; brown: overlap of *Cr. schmidti* and *Cr. ionia* s.l.

Our visual presentation of empirical color data shows that the mean coloration of *Ca. lateralis* differed throughout the study range and often resembled the color pattern of the regional *Crematogaster* model (Fig. 5). *Crematogaster scutellaris* had a reddish head and a blackish rest of the body; the *scutellaris*-syntopic category of *Ca. lateralis* had, in contrast, on average a more brownish instead of reddish head while the mesosoma was often lighter, compared to its model. Between the Dalmatian Peninsula Pelješac and the northwest of the Peloponnese, both model and mimic shared are a reddish head, mesosoma, and petiole. Model and mimic had dark brownish heads and mesosomas in the east of the Peloponnese and on Karpathos, while both were blackish on Crete.

## Research question 3: Can *Camponotus lateralis* be correctly allocated to the syntopic model species using color values, and does classification accuracy differ among model species?

A cross-validation linear discriminant analyses (LDA) using color values of *Crematogaster* workers yielded 98.3%, 97.5%, and 96.7% correctly-classified individuals in the three species (Table 1). The LDA suggested nine characters for classification: sqrtR_he, sqrtG_he, sqrtR_pr, sqrtG_pr, sqrtR_me, sqrtR_pp, sqrtG_pp, sqrtR_pe, and sqrtR_ga (definitions in Methods). Allocating *Ca. lateralis* workers as wild cards to the RGB data of the three *Crematogaster* species led to 66.0%, 98.5%, and 88.0% of correctly classified cases, respectively, in *scutellaris*-syntopic, *schmidti*-syntopic, and *ionia*-syntopic workers (Table 2). The *scutellaris*-syntopic workers had a strong tendency towards *Cr. schmidti* (29.6%).

**Table 1:** Correctly classified cases of *Crematogaster* workers of a cross-validation LDA using color variables.

*Crematogaster* species	predicted group membership		
	*scutellaris*	*schmidti*	*ionia* s.l
*scutellaris* (*n* = 179)	98.3%	1.1%	0.6%
*schmidti* (*n* = 244)	0.0%	97.5%	2.5%
*ionia* s.l. (*n* = 150)	0.0%	3.3%	96.7%

**Table 2:** Wild-card allocation of *Camponotus lateralis* minor-workers of the three syntopy categories, using color variables of the three *Crematogaster* model-species as calibration data.

*Ca. lateralis* minor-workers			predicted group membership		
site	syntopic model	*n*	*scutellaris* (%)	*schmidti* (%)	*ionia* s.l. (%)
1	*scutellaris*	43	81.4	16.3	2.3
2	*scutellaris*	82	79.3	20.7	0.0
3	*scutellaris*	42	78.5	16.7	4.8
4	*schmidti*	39	0.0	100.0	0.0
5	*scutellaris*	31	25.8	67.7	6.5
6	*scutellaris*	110	60.9	29.0	9.0
7	*scutellaris*	40	57.5	40.0	2.5
8	*scutellaris*	40	62.5	32.5	5.0
9	*schmidti*	73	0.0	100.0	0.0
10	*schmidti*	60	0.0	96.7	3.3
11	*schmidti*	65	0.0	100.0	0.0
12	*schmidti*	50	0.0	94.0	6.0
13	*schmidti*	40	0.0	100.0	0.0
14	*ionia* s.l	50	4.0	14.0	82.0
15	*ionia* s.l	52	1.9	0.0	98.1
16	*ionia* s.l	40	5.0	0.0	95.0
17	*ionia* s.l	50	2.0	0.0	98.0
18	*ionia* s.l	50	0.0	36.0	64.0
all *scutellaris* sites		388	66.0	29.6	4.4
all *schmidti* sites		327	0.0	98.5	1.5
all *ionia* s.l. sites		242	2.5	9.5	88.0

**Fig. 6:**
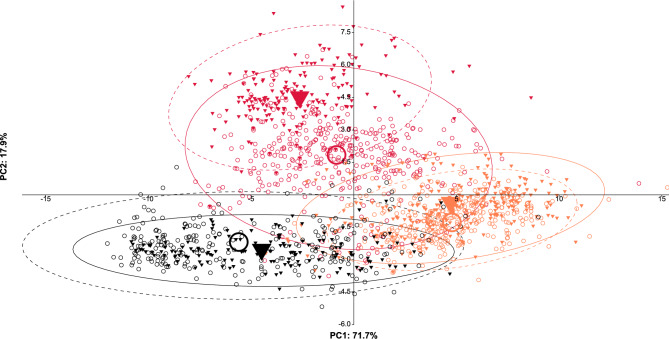
PCA of ant individuals using the nine color variables selected by the LDA (sqrtR_he, sqrtG_he, sqrtR_pr, sqrtG_pr, sqrtR_me, sqrtR_pp, sqrtG_pp, sqrtR_pe, sqrtR_ga). Red triangles: *Crematogaster scutellaris*; red circles: *scutellaris*-syntopic *Camponotus lateralis*; orange triangles: *Cr. schmidti*; orange circles: *schmidti*-syntopic *Ca. lateralis*; black triangles: *Cr. ionia* s.l.; black circles: *ionia*-syntopic *Ca. lateralis*. Means are represented by large symbols. Confidence intervals have been set at 95%, in *Crematogaster* they are dashed.

**Fig. 7:**
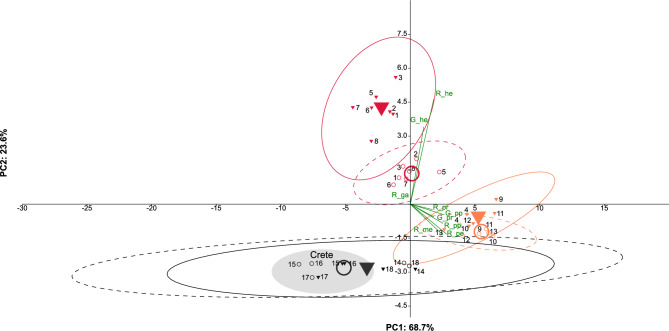
PCA of site means. Red triangles: *Crematogaster scutellaris*; red circles: *scutellaris*-syntopic *Camponotus lateralis*; orange triangles: *Cr. schmidti*; orange circles: *schmidti*-syntopic *Ca. lateralis*; black triangles: *Cr. ionia* s.l.; black circles: *ionia*-syntopic *Ca. lateralis*. Means are represented by large symbols. Confidence intervals have been set at 95%, in *Crematogaster* they are dashed. The collection sites on Crete are highlighted. The green vectors represent loadings of the nine color variables in the PCA space.

Principal component analyses (PCAs) using the reduced character set suggested by the LDA showed a large overlap of *Cr. schmidti* and *Cr. ionia* with the respective syntopically occurring mimics, while the *scutellaris*-syntopic category of *Ca. lateralis* was placed intermediately between all three model species, with the strongest tendency towards *Cr. scutellaris* and the weakest to *Cr. ionia* s.l. (Figs. 6, 7).

The PCA of site means showed a clear separation of the *Ca. lateralis* categories and they lay closely to their respective models (Fig. 7). Model and mimic from the three Cretan sites cluster closer together with each other than with populations from the two other sites of *Cr. ionia* s.l. The two head variables co-vary most tightly with each other and with *Cr. scutellaris* and its mimic; color variables of the mesosoma and petiole co-vary with each other and with *Cr. schmidti* and its mimic; the gaster variable shows no strong effect (loadings in Fig. 7).

## Discussion

### *Camponotus lateralis* mimics the color of three model species

The results showed that the three *Camponotus lateralis* categories—defined based on their syntopic *Crematogaster* model-species—differ in coloration of head, pronotum, mesonotum, propodeum, and petiole (Fig. 1). This suggests the existence of distinct regional color morphs instead of a general interspecies coloration strategy.

Moreover, Euclidean distances (Fig. 3), LDA wild-card results (Table 2), and PCAs (Figs. 6, 7) of color values showed that *Ca. lateralis* workers can not only be categorized geographically but also resemble the color patterns of local model species (Fig. 5). Hence, the data fully support the hypothesizes of regional Batesian color mimicry. While *Ca. lateralis* mimics *Cr. schmidti* and *Cr. ionia* s.l. impressively accurately in their range, the *scutellaris*-syntopic category, in contrast, mimics *Cr. scutellaris* least accurately. From the latter, nearly a third of individuals resembled the color pattern of the allotopic species *Cr. schmidti* instead.

### Why is *Crematogaster scutellaris* mimicked worse than other models?

Although all three *Camponotus lateralis* categories mimicked syntopic *Crematogaster* species, a tendency for the *scutellaris*-syntopic category to mimic the allopatric model *Cr. schmidti* was observed throughout the statistical analyses. Additionally, the visual comparison (Fig. 5) showed that the *scutellaris*-syntopic category failed to imitate the conspicuous color contrast between reddish head and blackish mesosoma of *Cr. scutellaris* accurately. One could argue that this inaccuracy is an edge effect caused by the small geographic distance to the range of *Cr. schmidti* and that results from a broader geographic range would differ. However, many pictures from Iberia and France (https://www.inaturalist.org/) also showed *Ca. lateralis* with a reddish mesosoma, suggesting *schmidti*-like *Ca. lateralis* workers occur over the whole range of *Cr. scutellaris*.

Inaccurate ant mimicry has frequently been observed and there are multiple hypotheses explaining this phenomenon^26^; it can be the result of tradeoffs, where mimicry with greater accuracy results in other costs^34,35^, or an adaption to mimic multiple model species at once^31,32^. It could also be the result of relaxed selection, occurring after the resemblance has reached a certain threshold, where further accuracy yield no additional benefit^26,29,36^. However, in cases described in literature, the Batesian mimic was never an ant, but another arthropod taxon. Hence, it is questionable to which extent such explanations are applicable to ant-ant mimicry systems. For example, morphological constraints—suggested to inhibit accurate mimicry in ant-mimicking spiders^33,36^—may play only a minor role in ant-ant mimicry due to ancestral shape similarity of model and mimic. In hoverflies, which mimic hymenopterans often inaccurately^21,94^, there can be physiological trade-offs: Dark-colored hoverflies are less accurate mimics but are better adapted to thermoregulation in temperate regions^95^. None of these concepts would fully explain why the *schmidti-*syntopic and the *ionia-*syntopic categories of *Ca. lateralis* are more accurate. Physiological constraints limiting *Ca. lateralis* from producing a *scutellaris*-like head-mesosoma contrast are unlikely, since some *scutellaris*-syntopic workers displayed this pigmentation pattern very well. We suggest to explain the observed case of inaccurate mimicry in the *scutellaris*-syntopic category by one or both of the following scenarios:

1. The evolutionary older models of *Ca. lateralis* were *Cr. schmidti* and *Cr. ionia* s.l. *Camponotus lateralis* populated the Western Mediterranean later, and a high percentage of *schmidti*-like workers still occurs there (due to relaxed selection?). An East Mediterranean origin of *Ca. lateralis* would be in line with our preliminary molecular-genetic data (Wagner et al. in prep.) as well as with the fact that seven of eleven species of the *Ca. lateralis* group occur only east of Italy. For example, the species which is most similar to *Ca. lateralis*, *Ca. anatolicus*, is restricted to Anatolia^66,68^. Only three species of the group, *Ca. lateralis*, *Ca. piceus*, and *Ca. spissinodis* have migrated to the Apennine Peninsula, Iberia, or NW Africa^66^. Alternatively, it seems also possible that the color pattern of the model itself, *Cr. scutellaris*, emerged in recent evolutionary times and *Ca. lateralis* still lags behind in resembling its color pattern. Inaccurate mimicry traits might be replaced by accurate ones in the evolutionary future, if selection pressure by predators is strong enough^96^.
2. In *Cr. scutellaris* and *Cr. schmidti*, stochastic shifts of distribution margins over evolutionary timescales might have led to ambiguity regarding which model is to be mimicked. In such cases of mimicking two model species at once, it may be the better compromise to present an overly large reddish portion rather than a too small one: Maybe any predators assess *Crematogaster*-sized ants to be more unpalatable, the more reddish they are. If so, it could prove better to resemble *Cr. schmidti* (having a reddish mesosoma) in syntopy with *Cr. scutellaris*, than conversely, to resemble *Cr. scutellaris* (having a blackish mesosoma) in syntopy with *Cr. schmidti*. To stand out from the majority of ant species outside the mimicry system being blackish, *schmidti*-like *Ca. lateralis* workers in syntopy with *Cr. scutellaris* tend to surpass the reddish warning signal of their model. This reasoning is similar to the concept of supernormal stimuli (with the difference that the rejection of the reddish color by predators is not inherent), which exceed natural ones and thus elicit a stronger behavioral response by the receiver^97,98^. However, the head in the *scutellaris*-syntopic category is less reddish and thus less conspicuous than in *Cr. scutellaris* (Fig. 5), which points against the idea of a “supernormal stimulus”. Nevertheless, it remains plausible that *schmidti*-like *Ca. lateralis* workers—which are accurate mimics in the range of *Cr. schmidti*—are more likely to deceive predators in syntopy with *Cr. scutellaris* than *scutellaris*-like ones in syntopy with *Cr. schmidti*.

### Regional mimicry also affects body size

Apart from color mimicry, we also detected weak regional body-size mimicry; *Crematogaster scutellaris* had a significantly larger cephalic size than the other two investigated *Crematogaster* species—a finding which is in line with data provided by Seifert^8^. The tendency of *scutellaris*-syntopic minor workers of *Ca. lateralis* to have a larger size than others was not significant (Fig. 2). However, average size differences between nests of *Crematogaster* model-species and nests of *Camponotus lateralis* were slightly significant smaller in syn- than in allotopy (Fig. 4). In addition to color mimicry, it may be adaptive not to stand out in size from *Crematogaster* workers to deceive potential predators. However, even though mostly minor workers are seen on *Crematogaster* trails, while major workers often stay in the nests, the occasional trail following of majors^53,57^ challenges this hypothesis. Although the species of the *Cr. scutellaris* group are monomorphic^8^, size can vary intranidally. Hence, the mimetic selection pressure on size may be considerably lower than that on color^99^.

The size difference between *Cr. scutellaris* and the two other *Crematogaster* species is ca. three times larger than between the *scutellaris*-syntopic category of *Ca. lateralis* and the two other categories. It shows that the differentiation between the *Crematogaster* species is stronger than between the *Ca. lateralis* categories not only in color but also in size, giving the impression that the mimic lags behind its models in phenotypic evolution.

### Repeated color-morph evolution in *Crematogaster* models and camponotine mimics

There are, meanwhile, several examples of camponotine ants mimicking other ants^16,40,45,46,100^. Among them, the Canarian example of *Camponotus guanchus* mimicking two color morphs of *Crematogaster alluaudi*, is particularly remarkable, as its two color morphs, one with a blackish and one with a reddish mesosoma, resemble the color patterns of *Cr. scutellaris* and *Cr. schmidti* and their mimics^46^. The case of Mediterranean *Colobopsis* species is similar: *Colobopsis truncata* resembles the color pattern of the widespread *Dolichoderus quadripunctatus*, its sister species *Co. imitans* mimics *Cr. scutellaris*^45^. These fascinating examples of analogous evolution highlight the adaptiveness of mimicking regional *Crematogaster* models.

The analogy with *Cr. alluaudi* also raises questions regarding the repeated evolution of aposematic color differentiation in the models. This differentiation should be evolutionarily older than regional mimetic traits in *Camponotus*. Our cross-validation LDA showed that individuals of the three different *Crematogaster* species were in nearly all cases correctly classified (Table 1); this indicates that color variables possess strong discriminant power. Similarly, in *Cr. alluaudi* intermediate forms between the two color morphs are unknown^46^. Such strong color differences enabling safe species delimitation are unusual for ants^8,101,102^, indicating that specific selection pressure may underlie this phenomenon^103^. The fact that species of the *Cr. scutellaris* group and color morphs of *Cr. alluaudi* exhibit color patterns, which are easy to distinguish from each other, prompts to speculate that color tends to evolve away from the ancestral state and thus also away from the color scheme of the mimics. Considering that mimicry increases predation risk for models due to diluting the honesty of the aposematic signal^18^, the pressure promoting this model radiation might be chase-away selection^104^—a force so far only known from aggressive^105^ but not from Batesian mimicry^26,106^.

## Material and methods

### Sampling

Nest samples of the *Crematogaster scutellaris* group and *Camponotus lateralis* were collected at 18 localities from Lombardy Italy (near Iseo Lake) across the Balkans to Karpathos, about ca. 1800 km linear distance (Supplementary material Table S5). A total of 179, 244, 150, and 957 workers were used from 24, 25, 15, and 101 colonies of *Cr. scutellaris*, *Cr. schmidti*, *Cr. ionia* s.l., and *Ca. lateralis*, respectively. Per locality, 20–60 (mean: 32) workers from 3–6 nests of *Crematogaster* and 37–111 (53) workers from 3–9 nests of *Ca. lateralis* were used.

### Color and size measurements

In *Camponotus lateralis*, only minor workers were used for color and size measurements. Ants were mounted on white paper cards. Dust particles on the body surface were removed with a point of the needle under a Wild Heerbrugg microscope with a magnification of 50x. To minimize effects of stray light, the photographs were taken in a room without window. Before taking the photographs, a white balance was performed (Results of the white balance: Red 617, Green 256, and Blue 338). Photographs of head, mesosoma (incl. petiole), and gaster were taken separately from dorsal view. Using a pin-holding stage, the head was tilted to the position with maximal cephalic length and width in visual plane. The mesosoma was tilted in dorsal view with pronotal neck and dorsalmost point of propodeum at the same focal level. Approximately 15–20 images were taken in different focal planes (distance 25 μm) with a Keyence VHX-5000 digital microscope and a Keyence VH Z100R Real Zoom Lens. As light source, a Keyence OP-87792 ring light was used (exposure time: 24 ms). The images were stacked automatically and a scale according to the magnification generated.

**Fig. 8:**
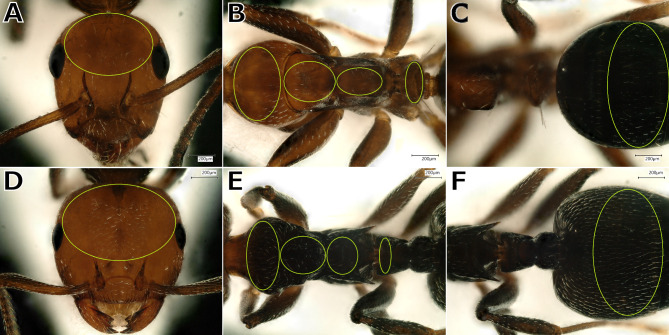
Body regions of which the average RGB values were taken; (**A**–**C**) *Camponotus lateralis*; (**D**–**F**) *Crematogaster* spp.; body regions from left to right: head (**A**,**D**), pronotum (**B**,**E**), mesonotum (**B**,**E**), propodeum (**B**,**E**), petiole (**B**,**E**), and gaster (**C**,**F**).

Mean RGB values of six body regions (Fig. 8), cephalic length, and cephalic width sensu Seifert^8^ (Table 3) were measured using ImageJ^107^ (Supplementary material Table S6).

**Table 3:** Acronyms and definitions of the worker RGB and morphometric characters.

Acronym	Definition
CL	Maximum cephalic (head) length in median line; the head must be carefully tilted to the position with the true maximum. Excavations of hind vertex and/or clypeus reduce CL^8^
CS	Cephalic (head) size; the arithmetic mean of CL and CW, used as a less variable indicator of body size^8^
CW	Maximum measurable cephalic (head) width. The position of measuring line is defined alone by the maximum and may be across or behind the eyes, varying between the genera considered^8^
R_he, G_he, B_he	Mean RGB values of head. Measured in an ellipsis touching the median occiput, the caudo-dorsal margin of both eyes, and caudalmost endpoints of dorsal carinae (Fig. 8: A, D)
R_pr, G_pr, B_pr	Mean RGB values of dorsum of pronotum measured in an ellipsis touching the dorsofrontal corner of pronotal slope, dorsolateral corners of pronotal slope, and anterior margin of suture to mesonotum (Fig. 8: B, E)
R_me, G_me, B_me	Mean RGB values of dorsum of mesonotum measured in an ellipsis touching the caudal margin of suture to pronotum and anterior margin of metanotal groove. Enlarge the ellipsis laterally until lateral slopes or margins of mesonotal spiracles (Fig. 8: B, E). Dark lateral parts (e.g., strong rugae in *Crematogaster*) of propodeal slope must not be inside the ellipsis
R_pp, G_pp, B_pp	Mean RGB values of dorsum of propodeum measured in an ellipsis touching caudal margin of metanotal groove, lateral corners of propodeal slope, and posterior margin dorsal propodeal plane (*Camponotus*) or suture to metapleuron (*Crematogaster*) (Fig. 8: B, E). Since the latter is not visible in *Crematogaster*, the point is defined to be between frontalmost points of propodeal spine declivities. Dark lateral parts of propodeal slope must not be inside the ellipsis
R_pe, G_pe, B_pe	Mean RGB values of dorsum of petiole measured in an ellipsis touching slopes frontal, lateral, and caudal (Fig. 8: B, E). Dark lateral parts of propodeal slope must not be inside the ellipsis
R_ga, G_ga, B_ga	Mean RGB values of gaster: In *Camponotus* an ellipsis over whole dorsum of second gastral tergite (Fig. 8: C), in *Crematogaster* an ellipsis over last two thirds of the first gastral tergite (Fig. 8: F)

### Statistics

Since RGB raw-data distribution was right-skewed, square roots (abbreviated as “sqrt”) of each of the 18 color variables were used for all analyses (using square roots is usual also in analyses of morphometric data^8^). Analyses to answer three research questions were performed:

## Research question 1: Which color or size traits differ between the three *Crematogaster* model-species and between their syntopic* Camponotus lateralis* mimics?

RGB data of each body part of each individual were reduced to PC1 using PAST 4.13^108^. Each color PC1 and size variable of individuals was analyzed via generalized linear mixed models using the lmer() function from the lme4 package^109^ in R^110^ to test putative differences in *Crematogaster* species as well as *Camponotus lateralis* categories. Thereby, species (in *Crematogaster*) or categories (in *Camponotus*) were used as fixed effect; nests and sites were used as random factors, with ‘nest’ nested within ‘site’. The global alpha-level of 0.05 was corrected using the Bonferroni-Holm method^111^.

## Research question 2: Is *Camponotus lateralis* more similar to syn- than to allotopic models in color and size?

Nest-mean data were reduced to principal components that explained cumulatively at least 80% of variance. Using PAST, Euclidean distances between nest means were calculated. Then, for each site, the mean Euclidean color distances between each *Ca. lateralis* nest-mean to each syntopic *Crematogaster* nest-mean were calculated. To test if *Ca. lateralis* also mimics size, cephalic size differences between each *Ca. lateralis* nest-mean to each syntopic *Crematogaster* nest-mean were calculated. The means of the 17 allotopic Euclidean distances or size differences per site were each calculated and then averaged. Finally, a one-sided type-1 t-test^112^ was applied to test if syntopic Euclidean distances or size differences were smaller than allotopic ones.

For a visual presentation of color congruence between *Crematogaster* and *Ca. lateralis* occurring in the same regions, the empirical means of the 18 color variables for each site were calculated and implemented into a map. To obtain the color, the raw R, G, and B values of the individuals of each genus per site for each body part were averaged. For the mesosoma, the mean R, G, and B values of all three mesosomal regions were taken. The map was made using QGIS 3.42.2 (QGIS Development Team, 2024) with resources taken from Natural Earth (naturalearthdata.com).

## Research question 3: Can *Camponotus lateralis* be correctly allocated to the syntopic model species using color values, and does classification accuracy differ among model species?

A linear discriminant analysis (LDA), using all 18 RGB square-root-transformed variables, was performed in SPSS Statistics Version 30 (IBM, USA) using default settings and the stepwise method. It was important to use solely data of *Crematogaster* for the calibration, because *Crematogaster* was the model. Affiliation of *Crematogaster* individuals was evaluated via a cross validation. The individuals of the three mimetic *Ca. lateralis* categories were used as wild cards^113^ in order to evaluate the percentages of correctly classified individuals to each syntopic *Crematogaster* model-species.

Using the reduced character set suggested by the LDA (sqrtR_he, sqrtG_he, sqrtR_pr, sqrtG_pr, sqrtR_me, sqrtR_pp, sqrtG_pp, sqrtR_pe, sqrtR_ga), PCAs were performed in PAST for each *Crematogaster* species and *Ca. lateralis* category on the levels of individuals and sites, and visualized as scatter plots. Ellipses showing 95% confidence intervals were implemented into the figures automatically.

DeepL was used to revise the manuscript linguistically.

## Supplementary Information

Supplementary Information 1.

Supplementary Information 2.

## Data Availability

All data generated or analyzed during this study are included in this published article and its supplementary information (Supplementary Material Tabs S1–S6).
