# Supplementary material for: Evolutionary arms race in ant-ant mimicry: Camponotus lateralis lags behind in mimicking color patterns and sizes of regional Crematogaster models
Source: Sci Rep. 2025 Nov 20;15:41076. doi: 10.1038/s41598-025-25035-y (PMC12635149; doi:10.1038/s41598-025-25035-y)
Supplement: Supplementary file 1 — Supplementary Information 1. [file 41598_2025_25035_MOESM1_ESM.docx]

**Evolutionary arms race in ant-ant mimicry: *Camponotus lateralis* lags behind in mimicking color patterns and sizes of regional *Crematogaster* models**

**Felix Kraker & Herbert C. Wagner**

Table S1: Average values and standard deviation of the square roots of the 18 RGB variables of the three *Crematogaster* model-species and the three *Camponotus lateralis* categories.

|  | *Crematogaster scutellaris* sites | | *Crematogaster schmidti* sites | | *Crematogaster ionia* s.l. sites | |
| --- | --- | --- | --- | --- | --- | --- |
|  | *scutellaris* | *lateralis* | *schmidti* | *lateralis* | *ionia* s.l. | *lateralis* |
| *n* | 179 | 388 | 244 | 327 | 150 | 242 |
| sqrtR_he | 11.29 ± 1.28 | 9.68 ± 1.45 | 9.86 ± 1.21 | 9.35 ± 1.12 | 5.43 ± 1.41 | 5.27 ± 1.62 |
| sqrtG_he | 8.14 ± 1.26 | 6.56 ± 1.10 | 6.87 ± 1.08 | 6.13  ± 0.93 | 3.99 ± 0.73 | 3.64 ± 0.9 |
| sqrtB_he | 4.24 ± 0.74 | 3.46 ± 0.55 | 3.90 ± 0.60 | 3.50 ± 0.47 | 3.13 ± 0.26 | 2.82 ± 0.29 |
| sqrtR_pr | 6.10 ± 1.30 | 8.28 ± 1.62 | 9.26 ± 1.43 | 10.52 ± 0.92 | 5.80 ± 1.45 | 6.25  ± 2.10 |
| sqrtG_pr | 4.70 ± 0.87 | 5.69 ± 1.10 | 6.71 ± 1.15 | 7.45 ± 0.97 | 4.47 ± 0.83 | 4.44 ± 1.28 |
| sqrtB_ pr | 3.66 ± 0.52 | 3.55 ± 0.44 | 4.17 ± 0.50 | 4.17 ± 0.60 | 3.48 ± 0.34 | 3.3 ± 0.38 |
| sqrtR_me | 5.8 ± 1.29 | 8.23 ± 1.52 | 9.35 ± 1.52 | 10.3 ± 1.08 | 6.24 ± 1.63 | 6.23 ± 1.80 |
| sqrtG_me | 4.79 ± 0.86 | 5.89 ± 0.95 | 6.87 ± 1.25 | 7.39 ± 0.96 | 4.89 ± 0.97 | 4.81 ± 0.98 |
| sqrtB_me | 4.08 ± 0.61 | 4.21 ± 0.53 | 4.63 ± 0.59 | 4.72 ± 0.58 | 3.92 ± 0.44 | 3.95 ± 0.36 |
| sqrtR_pp | 5.73 ± 1.02 | 7.09 ± 1.58 | 10.44 ± 1.14 | 10.1 ± 0.99 | 6.76 ± 1.70 | 5.83 ± 2.00 |
| sqrtG_pp | 4.95 ± 0.70 | 5.51 ± 0.92 | 7.94 ± 1.10 | 7.43 ± 0.94 | 5.25 ± 1.01 | 4.56 ± 1.17 |
| sqrtB_pp | 4.32 ± 0.62 | 4.13 ± 0.51 | 5.28 ± 0.72 | 4.68 ± 0.68 | 4.03 ± 0.49 | 3.53 ± 0.40 |
| sqrtR_pe | 6.31 ± 1.18 | 5.99 ± 1.43 | 10.64 ± 1.00 | 9.50 ± 1.25 | 7.14 ± 2.16 | 6.15 ± 1.82 |
| sqrtG_pe | 5.37 ± 0.78 | 4.86 ± 0.90 | 8.08 ± 1.02 | 7.07 ± 1.11 | 5.59  ± 1.37 | 4.75 ± 1.15 |
| sqrtB_pe | 4.41 0.73 | 3.65 ± 0.48 | 5.19 ± 0.79 | 4.37 ± 0.78 | 4.15 ± 0.72 | 3.41 ± 0.43 |
| sqrtR_ga | 5.33 ± 0.90 | 3.94 ± 0.59 | 5.28 ± 0.62 | 4.28 ± 0.67 | 5.44 ± 0.86 | 4.14 ± 0.71 |
| sqrtG_ga | 5.11  ± 0.65 | 4.02 ± 0.60 | 5.13 ± 0.53 | 4.24 ± 0.62 | 4.88 ± 0.48 | 3.83 ± 0.50 |
| sqrtB_ga | 4.50 ± 0.64 | 3.58 ± 0.54 | 4.57 ± 0.52 | 3.81 ± 0.60 | 4.11 ± 0.32 | 3.32 ± 0.41 |

Table S2: Pairwise *p*-values for comparisons of the first principal components of color traits for six body parts between *Crematogaster scutellaris* (*n* = 179), *Cr. schmidti* (*n* = 244), and *Cr. ionia* (*n* = 150) as well as between the *scutellaris*-syntopic (*n* = 388), the *schmidti*-syntopic (*n* = 327), and the *ionia*-syntopic (*n* = 242) *Camponotus lateralis* categories analyzed via generalized linear mixed models. Significant results after Bonferroni-Holm correction are shown in **bold**.

|  | *Cr. scutellaris* vs. *schmidti* sites | | *Cr. scutellaris* vs. *ionia* sites | | *Cr. schmidti* vs. *ionia* sites | |
| --- | --- | --- | --- | --- | --- | --- |
| body parts | *Crematogaster* | *Ca. lateralis* | *Crematogaster* | *Ca. lateralis* | *Crematogaster* | *Ca. lateralis* |
| head | 0.0059 | 0.5825 | **< 0.0001** | **< 0.0001** | **< 0.0001** | **< 0.0001** |
| pronotum | **< 0.0001** | **0.0002** | 0.3140 | 0.0086 | **< 0.0001** | **< 0.0001** |
| mesonotum | **< 0.0001** | **0.0001** | 0.9445 | 0.0041 | **< 0.0001** | **< 0.0001** |
| propodeum | **< 0.0001** | **0.0001** | 0.4975 | 0.0103 | **< 0.0001** | **< 0.0001** |
| petiole | **< 0.0001** | **< 0.0001** | 0.6581 | 0.7052 | **0.0001** | **< 0.0001** |
| gaster | 0.8148 | 0.1961 | 0.4935 | 0.4918 | 0.3782 | 0.0737 |

Table S3: Average Euclidean color distances between nests of *Crematogaster* model-species and of *Camponotus lateralis*.

| site | syntopic Euclidean color distances | allotopic Euclidean color distance |
| --- | --- | --- |
| 1 | 4.428 | 5.829 |
| 2 | 3.872 | 6.065 |
| 3 | 5.167 | 5.949 |
| 4 | 3.295 | 7.660 |
| 5 | 6.191 | 6.945 |
| 6 | 3.997 | 6.124 |
| 7 | 6.773 | 6.594 |
| 8 | 3.815 | 6.000 |
| 9 | 2.911 | 8.071 |
| 10 | 3.536 | 9.483 |
| 11 | 1.925 | 8.111 |
| 12 | 3.503 | 7.321 |
| 13 | 3.913 | 8.387 |
| 14 | 2.361 | 6.687 |
| 15 | 3.907 | 10.906 |
| 16 | 3.473 | 10.274 |
| 17 | 2.386 | 10.107 |
| 18 | 2.396 | 6.826 |
| **Mean** | 3.77 | 7.63 |
| **Stabw** | 1.28 | 1.63 |
| ***p* value** | 1.3134E^-6^ |  |

Table S4: Average size differences between nests of *Crematogaster* model-species and of *Camponotus lateralis*.

| site | syntopic size differences [μm] | allotopic size differences [μm] |
| --- | --- | --- |
| 1 | 39.17 | 68.78 |
| 2 | 86.79 | 71.92 |
| 3 | 128.87 | 91.61 |
| 4 | 75.21 | 87.47 |
| 5 | 68.81 | 92.49 |
| 6 | 61.25 | 63.88 |
| 7 | 106.63 | 89.34 |
| 8 | 89.35 | 86.68 |
| 9 | 66.55 | 84.32 |
| 10 | 54.46 | 57.15 |
| 11 | 75.65 | 80.67 |
| 12 | 30.07 | 62.13 |
| 13 | 71.47 | 76.17 |
| 14 | 62.20 | 79.90 |
| 15 | 56.28 | 67.96 |
| 16 | 62.42 | 76.84 |
| 17 | 66.24 | 89.59 |
| 18 | 30.73 | 66.98 |
| **Mean** | 68.45 | 77.44 |
| **Stabw** | 24.52 | 11.12 |
| ***p* value** | 0.0289 |  |

Table S5: Overview about the 18 sampling localities.

| site | locality | coordinates | habitat | model | date | collector |
| --- | --- | --- | --- | --- | --- | --- |
| 1 | Italy: 1.4 km WNE Passirano | 45.6032° N, 10.0477° E, 252 m | mixed-deciduous-forest margin | *Cr. scutellaris* | 24.-25.IV.2023 | H. C. Wagner |
| 2 | Italy: 1.2 km W Custoza | 45.3702° N, 10.7798° E, 109 m | deciduous-forest remnant | *Cr. scutellaris* | 22.-23.IV.2023 | H. C. Wagner |
| 3 | Italy: 1.1 km NNW Battaglia Terme | 45.2985° N, 11.7732° E, 14 m | *Morus* alley with *Hedera* *helix* and *Smilax* | *Cr. scutellaris* | 21.-22.IV.2023 | H. C. Wagner |
| 4a | Slovenia: 1.6 km NW Ankaran | 45.5908° N, 13.7234° E, 18 m | *Quercus*-*Ulmus* forest with *Hedera* *helix* | *Cr. schmidti* | 17.-19.IV. and 15.X.2023 | H. C. Wagner |
| 4b | Slovenia: 2.8 km NW Ankaran | 45.5913° N, 13.7042° E, 17 m | *Pinus* forest with *Hedera* *helix* | *Cr. schmidti* | 14.-15.X.2023 | H. C. Wagner |
| 5a | Croatia: 3.8 km ESE Novigrad | 45.3066° N, 13.6103° E, 69 m | meadow with *Quercus* trees with *Hedera* *helix* | *Cr. scutellaris* | 14.IV.2023 | H. C. Wagner |
| 5b | Croatia: 3.3 km ESE Novigrad | 45.3113° N, 13.6057° E, 16 m | *Pinus* forest in north exposition | *Cr. scutellaris* | 15.IV.2023 | H. C. Wagner |
| 6 | Croatia: 1.7 km N Linardići (Krk) | 45.0889° N, 14.4667° E, 119 m | *Quercus*-*Ulmus* forest with *Hedera* *helix* | *Cr. scutellaris* | 12.-13.IV.2023 | H. C. Wagner |
| 7 | Croatia: 5.5 km NW Pirovac | 43.8476° N, 15.6123° E, 12 m | *Pinus* forest with *Hedera* *helix* and *Smilax* | *Cr. scutellaris* | 9.-10.IV.2023 | H. C. Wagner |
| 8 | Croatia: 2.2 km NNW Makaraska | 43.3151° N, 17.0020° E, 69 m | *Pinus* forest | *Cr. scutellaris* | 6.-7.IV.2023 | H. C. Wagner |
| 9 | Croatia: 9.5 km W Orobić | 42.9851° N, 17.0676° E, 27 m | Macchia with *Smilax* | *Cr. schmidti* | 4.-5.IV.2023 | H. C. Wagner |
| 10 | Montenegro: 3.8 km SE Petrovac | 42.1836° N, 18.9759° E, 5 m | *Quercus* trees with *Hedera* *helix* | *Cr. schmidti* | 2.-3.IV.2023 | H. C. Wagner |
| 11 | Albania: 8.0 km W Levan | 40.6748° N, 19.3940° E, 1 m | *Pinus* forest with *Pistacia* and *Quercus* bushes | *Cr. schmidti* | 26.-28.V.2023 | F. Kraker |
| 12 | Greece: 2.7 km SSE Ladochori | 39.4685° N, 20.2476° E, 9 m | deciduous forest | *Cr. schmidti* | 15.-16.IV.2024 | H. C. Wagner |
| 13 | Greece: 2.0 km SW Araxos | 38.1755° N, 21.3683° E, 2 m | *Quercus*-*Pistacia* forest with Smilax | *Cr. schmidti* | 12.-13.IV.2024 | H. C. Wagner |
| 14 | Greece: 2.2 km NNE Palea Epidauros | 37.6582° N, 23.1631° E, 8 m | *Pinus* forest with *Pistacia* shrubs | *Cr. ionia* s.l*.* | 9.-10.IV.2024 | H. C. Wagner |
| 15 | Greece: Crete, SW Lake Límni Agiás | 35.4747° N, 23.9316° E, 43 m | *Platanus* forest with *Hedera* *helix* | *Cr. ionia* s.l. | 4.-5.IV.2024 | H. C. Wagner |
| 16 | Greece: Crete, 1.4 km WSW Argiroupolis | 35.2822° N, 24.3191° E, 180 m | deciduous wood along river | *Cr. ionia* s.l. | 2.-3.IV.2024 | H. C. Wagner |
| 17 | Greece: Crete, 0.8 km W Maronia | 35.1404° N, 26.0728° E, 222 m | humid *Eucalyptus*-*Platanus* ditch with *Hedera* *helix* and *Ficus* | *Cr. ionia* s.l. | 29.-30.III.2024 | H. C. Wagner |
| 18 | Greece: Karpathos, 5.7 km N Spoa | 35.6877° N, 27.1504° E, 192 m | *Pinus* forest in north exposition | *Cr. ionia* s.l. | 26.-27.III.2024 | H. C. Wagner |
